# New Insights on Singlet Oxygen Release from Li-Air Battery Cathode: Periodic DFT Versus CASPT2 Embedded Cluster Calculations

**DOI:** 10.1021/acs.jctc.3c00393

**Published:** 2023-07-11

**Authors:** Francesca Fasulo, Arianna Massaro, Ana B. Muñoz-García, Michele Pavone

**Affiliations:** † Department of Physics “E. Pancini”, University of Naples Federico II, I-80126 Napoli, Italy; ‡ Department of Chemical Sciences, University of Naples Federico II, I-80126 Napoli, Italy; § National Reference Center for Electrochemical Energy Storage (GISEL)-INSTM, 50121 Florence, Italy

## Abstract

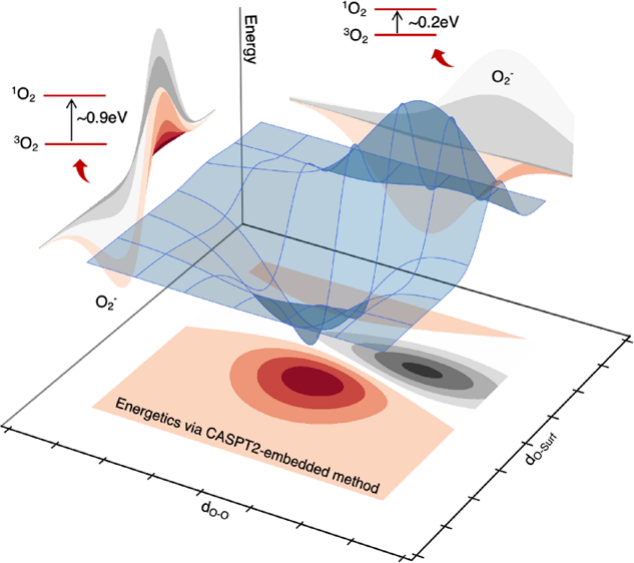

Li-air batteries are a promising energy storage technology for large-scale applications, but the release of highly reactive singlet oxygen (^1^O_2_) during battery operation represents a main concern that sensibly limits their effective deployment. An in-depth understanding of the reaction mechanisms underlying the ^1^O_2_ formation is crucial to prevent its detrimental reactions with the electrolyte species. However, describing the elusive chemistry of highly correlated species such as singlet oxygen represents a challenging task for state-of-the-art theoretical tools based on density functional theory. Thus, in this study, we apply an embedded cluster approach, based on CASPT2 and effective point charges, to address the evolution of ^1^O_2_ at the Li_2_O_2_ surface during oxidation, *i.e.*, the battery charging process. Based on recent hypothesis, we depict a feasible O_2_^2–^/O_2_^–^/O_2_ mechanisms occurring from the (112̅0)–Li_2_O_2_ surface termination. Our highly accurate calculations allow for the identification of a stable superoxide as local minimum along the potential energy surface (PES) for ^1^O_2_ release, which is not detected by periodic DFT. We find that ^1^O_2_ release proceeds *via* a superoxide intermediate in a two-step one-electron process or another still accessible pathway featuring a one-step two-electron mechanism. In both cases, it represents a feasible product of Li_2_O_2_ oxidation upon battery charging. Thus, tuning the relative stability of the intermediate superoxide species can enable key strategies aiming at controlling the detrimental development of ^1^O_2_ for new and highly performing Li-air batteries.

## Introduction

Development and exploitation of sustainable energy storage systems are pivotal topics we must address to solve the global energy crisis.^1^ Rechargeable batteries are expected to power many different applications, from portable electronics to large-scale electrical grids and electric vehicles.^2−5^ Since the work of Abraham in 1996, Li-air batteries represent a promising technology for automotive and electric transportation:^6−9^ enhanced specific capacity and low cell weight are their most attractive figures of merits, and the cathode is mainly composed by a lightweight porous material that hosts the gaseous O_2_ coming from the outside. The working principles rely on the oxygen reduction reaction (ORR) and oxygen evolution reaction (OER) occurring upon battery discharge and charge, respectively.^10^ During discharge, lithium metal (*i.e.*, the negative electrode) is oxidized to Li^+^, which diffuses through the electrolyte up to the porous material at the cathode side, where it reacts with the reduced oxygen species (ORR products). Conversely, upon charge, the reverse process occurs with restoration of starting materials (Li and O_2_).^11^

In principle, many discharge products can result from the ORR process, including lithium oxide, peroxide, and superoxide (*i.e.*, Li_2_O, Li_2_O_2_, and LiO_2_). Formation of Li_2_O is usually associated to formation of undesired insulating and nonmagnetic surfaces that may be the cause of the observed irreversibility.^12^ Thin layers resulting from Li_2_O_2_ production feature oxygen-rich composition with metallic and ferromagnetic character, whose facile pathways for electron transport clearly explain the corresponding higher electrochemical reversibility.^12,13^ Notably, electronic transport limitation through a passivating coating and mass transport of reactive species can sensibly hamper the Li_2_O_2_ capacity.^14^ The potential LiO_2_ discharge product is known to convert to the more stable Li_2_O_2_*via* a second electron transfer on the electrode surface or a disproportionation reaction in the solution, especially in high-donicity electrolytes that can lower the hardness of Li^+^ and favor the interaction with the soft superoxide base.^15,16^ From advanced characterization experiments, Li_2_O_2_ is considered the main ORR product, thus leading to the following cell reaction:^13,15^
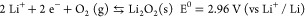
1Although eq 1 is theoretically reversible, the amount of gaseous O_2_ evolved during charge is typically less than 90% of that consumed during discharge, and the number of exchanged electrons per O_2_ in one full cycle deviates from ideality (2e^–^/O_2_).^17−19^

Clearly, the battery rechargeability directly depends on the efficiency of Li_2_O_2_ formation/decomposition cycle, and several research groups have been focusing on investigating this cycle for understanding its mechanistic landscape.^13,20^ The poor reversibility of Li-air batteries seems to be caused by degradation reactions mainly occurring upon charge. In 2016, remarkable results have suggested that the formation of singlet oxygen, namely, the first excited state of triplet dioxygen (^1^O_2_^1^:Δ_g_ → ^3^O_2_^3^:Σ_g_), plays a crucial role in the degradation of Li-air battery components.^21,22^ As a highly reactive oxidizing species, ^1^O_2_ might trigger irreversible parasitic reactions, thus undermining the overall battery performance and the long-term stability. By means of *in operando* electron paramagnetic resonance, Wandt *et al.* have detected the signal of a selective spin trapping agent forming a stable radical with ^1^O_2_, thus demonstrating that singlet oxygen is produced upon Li_2_O_2_ oxidation at potentials above 3.5 V.^21^ The comparative experiment performed by Mahne *et al.* proved the role of singlet oxygen in electrolyte degradation: the same side products typically observed upon Li_2_O_2_ oxidation (*i.e.*, the Li-air battery charge process), which mainly include lithium carbonate, acetate, and formate, have also been found when ^1^O_2_ is purposely and photochemically generated inside the cell.^22^ Despite these great advances, the origin of singlet oxygen is still under debate, and a comprehensive investigation of the elusive mechanism along its formation represents a challenging task for both experiments and theory.

Experimental evidences seem to agree in ascribing the ^1^O_2_ release to the electrochemical oxidation of Li_2_O_2_*via* a 2e^–^ oxidation process (reverse process in eq 1) at voltage values above 3.5 V.^21,23^ In addition, the higher ^1^O_2_ abundance detected upon charge over discharge consistently indicates that singlet oxygen mostly evolves from Li_2_O_2_ decomposition at increasing voltage.^22^ However, different discharge products can originate from ORR and more intricate reactivity scenarios may arise. As already anticipated, the fate of LiO_2_ eventually formed upon discharge involves either a disproportionation in solution or oxidation at the cathode surface, which would likely represent additional sources of ^1^O_2_.^24^ As a solution mechanism, the superoxide disproportionation is promoted in aprotic electrolytes with high donicity,^15^ where the solvent would strongly solvate the Li^+^ and leave the uncoordinated O_2_^–^ anions free to react:^25^
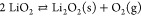
2While right-shifting the reaction equilibrium (eq 2) and favoring the solution-growth of Li_2_O_2_ with desired morphology, the solution mechanism in good electrolyte donors also yields large fractions of ^1^O_2_.^26,27^ Nevertheless, superoxide-like species may also derive from the one-electron oxidation of lithium peroxide upon charge (eq 3) and thus be involved in the release of singlet oxygen following a different reaction pathway (eq 4):
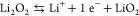
3
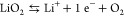
4While peroxide oxidation occurs at potential values of 2.96 V *vs* Li^+^/Li, lower voltage (but above ∼2.5 V, which is the standard potential of O_2_/O_2_^–^*vs* Li^+^/Li in aprotic solvents) would be sufficient for superoxide oxidation.^28−30^

As a general consideration, ^1^O_2_ release is both thermodynamically and kinetically unfavorable compared to ^3^O_2_ formation. The differences in the standard potentials for a reaction resulting in singlet or triplet oxygen are theoretically quantified as 0.96 and 0.48 V in the case of one and two transferred electrons, respectively (eqs 3–4 and 1).^31^ Singlet oxygen formation always happens when the Li-air battery reaches the required voltage able to overcome the otherwise more convenient reaction path toward the triplet state. As revealed by DFT studies, the superoxide disproportionation seems to be the dominant pathway toward ^1^O_2_, but redox mediators with potentials above 3.5 V *vs* Li^+^/Li can also drive the ^1^O_2_ evolution from peroxide/superoxide oxidation.^32^

Pursuing the fundamental understanding of such a complex reactivity framework is extremely helpful in this field, where the precise characterization of each mechanism and involved chemical species can assess specific solutions toward improved reversibility and longer cycle life. Successful strategies have been outlined to eliminate ^1^O_2_-induced side reactions, for example, by using ^1^O_2_ traps and quenchers as electrolyte additives.^22,33^ In this context, the role of theoretical chemistry is crucial, since the atomistic perspective coupled to accurate electronic structure analysis can provide insightful details on the origin, chemical nature, and reaction mechanisms behind ^1^O_2_ release. However, having reliable models for such tangled reactivity can be cumbersome, owing to the elusive features within the expected electronic states, including radicals in any open- or closed-shell configuration (*e.g.*, O_2_^–^, ^1^O_2_). Despite being widely employed, DFT is not suitable for these species due to intrinsic limitations in treating highly correlated systems.^34^ By incorporating both static and dynamic electron correlation effects, multireference approaches such as CASPT2 (complete active space plus second order perturbation theory) represent the methods of choice to ensure a reliable description of subtle electronic states in radical systems.^35−40^ A recent attempt to depict the singlet oxygen release at the CASPT2 level of theory has been reported by Zaichenko and co-workers.^41^ Therein, the authors have shown that the LiO_2_ disproportionation proceeds *via* two different paths over crossing points of different electronic states, leading either to the energetically preferred ^3^O_2_ or the energetically higher-lying (by 0.9 eV) ^1^O_2_ formation.^41^ By monitoring the oxygen–oxygen bond lengths and the oxygen–lithium distance along the electron-transfer process, the triplet state is shown to form at distance *d*_O–O,Li_ of ∼2.75 Å and associated to ∼2.5 eV, while the singlet oxygen can be released at ∼3.50 Å once provided an energy supply of ∼3.4 eV.^41^ In the work by Pierini *et al.*, gas-phase CASPT2 calculations on the LiO_2_^–^ anionic trimer show that the true nature of the electronic ground state is that of superoxide, which can only be unveiled by multiconfigurational methods.^42^ While a single-reference method would inevitably perceive the overall singlet multiplicity of the molecule as a closed shell arrangement of the electrons (thus yielding the M^+^O_2_^2–^ peroxide configuration), the multiconfigurational nature of an open-shell singlet diradical (*i.e.*, the M^0^(↑)O_2_^–^(↓) superoxide configuration) can only be distinguished with multireference approaches. The occurrence of this low-lying M^0^ superoxide channel in the possible products of superoxide disproportionation might have important consequences for the parasitic processes leading to cell death, for example, largely lowering the overpotentials needed to induce the ^1^O_2_ formation, which would explain why this reactive species has been observed despite the unfavorable energetics of superoxide oxidation.^42^

To the best of our knowledge, theoretical investigations on reaction mechanisms underlying singlet oxygen release have been reported only on small molecular systems in solution.^41,42^ Nevertheless, reaction energetics and involved species, as well as mechanistic details, can be largely different if the process takes place at the interface with solid-state particles. For example, the exposed crystalline facets on Li_2_O_2_ nanoparticles might act as reaction sites during the charging process, and the material surface can be key in the electron transfer occurring at the cathode boundary.^43^ The role of heterogeneous interfaces generated at the solid-state electrode/electrolyte contact should not be neglected. However, the use of multireference methods usually comes with increased computational costs, which definitely hinder their application to large systems, such as crystalline surfaces within periodic boundary conditions (PBC). Hence, we present a thorough study on ^3^O_2_/^1^O_2_ release from the (112̅0)-Li_2_O_2_ surface by means of an integrated computational approach based on electrostatically embedded-cluster CASPT2 methods.^44^ The success of electrostatic embedding approaches has been established for similar investigations aiming to unveil adsorption and reactivity processes on several material surfaces.^37−40^ By partitioning a given structural model in two subsystems featuring different chemical nature and properties, it is possible to design a cluster system, comprising the localized phenomena under study and the embedding environment, which in turn can affect the investigated properties. Defined this way, the electrostatically embedded-cluster (EC) model allows to treat the two partitions with different levels of theory, by refining the theoretical description with high accuracy to a localized portion (*e.g.*, applying the CASPT2 method to a small Li_2_O_2_ cluster containing the releasing dioxygen moiety) that is electrostatically embedded in a point charge (PC) array mimicking the extended surface (*e.g.*, employing a PC field for the (112̅0) Li_2_O_2_ surface slab). The choice of the (112̅0) lattice termination is motivated by the available literature reporting this crystal facet as one of the most abundant in Li_2_O_2_ nanoparticles under extreme reducing and oxidizing conditions.^45^ Comparison between embedded-cluster CASPT2 results (*i.e.*, EC(CASPT2-PC)) with periodic DFT (*i.e.*, PBC(PBE)) confirms the need of refined multireference calculations to accurately describe the elusive singlet oxygen state and to unveil the reaction mechanism for its release from the Li_2_O_2_ interface. The bond length of releasing oxygen moiety is used as a descriptor to predict the chemical nature of the evolved species, in line with previous reports.^41,42^ Our results show that singlet oxygen can develop from the Li_2_O_2_ surface according to two possible reaction pathways: (i) the two-step one-electron peroxide oxidation leading to a stable superoxide that acts as a reaction intermediate; (ii) the one-step two-electron peroxide oxidation passing through an unstable superoxide and still leading to ^1^O_2_. Both mechanisms feature energy barriers associated to bond reorganization and excitation to the higher-lying singlet state that can still become accessible under battery operating conditions, that is at the high voltage supplied upon charge. Embedded-cluster CASPT2 calculations performed in a charged model (*i.e.*, Li-defective surface slab) also show that ^1^O_2_ release can be favored when coupled to Li desorption. Overall, Li_2_O_2_ oxidation seems to be sensibly affected by the relative stability of superoxide species, whose fine-tuning can enable key strategies aiming to control the detrimental singlet oxygen development and thus assess the production of highly performing Li-air batteries.

## Methods and Computational Details

**Figure 1:**
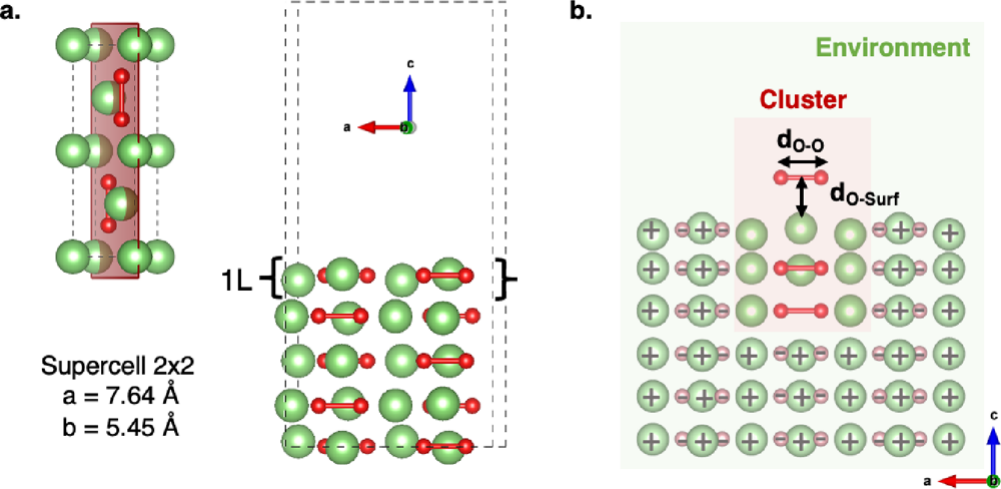
(a) Structural models of Li_2_O_2_ bulk unit cell and surface slab obtained by cleaving up the 1 × 1 × 1 supercell along the (112̅0) lattice direction. (b) Embedded-cluster model employed for CASPT2 calculations (highlighted in red): point charge field describing the environment (highlighted in green) and definition of reaction coordinates (oxygen–oxygen bond length, *d*_O–O_, and oxygen moiety–surface distance, *d*_O–Surf_). Atoms are represented as spheres. Color code: Li atoms (green), O atoms (red).

Spin-polarized density functional theory (DFT) calculations are performed within the supercell approach that employs periodic boundary conditions (PBC) and plane-wave (PW) basis sets, as implemented in the Vienna ab initio simulation package (VASP, ver. 5.4.1).^46−49^ We use the generalized gradient approximation (GGA) for the exchange-correlation functional proposed by Perdew, Burke, and Ernzerhof (PBE).^50,51^ Core electrons are described by projector-augmented wave (PAW) potentials obtained from the VASP repository, while the valence/outer-core electrons that are included in the self-consistent-field calculations are [1s^2^2s^1^] for Li and [2s^2^2p^4^] for O atoms.^52^ To build up the structural model for the (112̅0) surface, we apply the surface-slab approach by cleaving the optimized bulk structure along those lattice planes and then introducing 15 Å of vacuum along the c direction (see Figure 1a).^53^

The 2 × 2 supercell containing five layers is considered as a suitable structural model to achieve surface energy convergence (see Table S1 in the SI). Since we are dealing with asymmetric slabs with a net surface dipole density, dipole correction is taken into account as implemented in VASP code.^54^ Pseudo-wave functions are expanded in a PW basis set with a kinetic energy of 600 eV, and Γ-point mesh is used for sampling the Brillouin zone, as determined from energy convergence tests with 1 meV/f.u. threshold on the total electronic energy. We carry out geometry optimization of the surface slab by relaxing atomic positions until the maximum forces acting on each atom are below 30 meV/Å. To reproduce the desired spin multiplicity for the singlet/triplet oxygen states in the DFT calculations, we constrain the total spin moment at 0 and 2 to represent the closed-shell ^1^O_2_ and the open-shell ^3^O_2_ configurations, respectively.

The considered structural model for embedded-cluster CASPT2 calculations (Figure 1b) is a stoichiometric cluster of 4 Li_2_O_2_ formula units (f.u.) comprising the nearest 8 Li atoms and 4 O_2_ units (one of which is the releasing molecule) that is carved from the Li_2_O_2_ surface. The remaining surface slab represents the environment and is treated as a point charge array by means of the electrostatic embedding approach, as implemented in OpenMolcas.^36−40,55^ The supercell size is 7.64 Å × 8.28 Å × 8.49 Å and contains 729 Li (with +1 charge) and O (with −1 charge) point charges ensuring the electroneutrality of the system. The cc-PVTZ basis set is employed on each atom, while effective core potentials (ECPs) with no electrons are adopted for Li^+^ cation lying in the first coordination shell surrounding the cluster in order to avoid artificial drift of electron density onto nearby positive point charges.^56^ This large basis set ensures a basis set superposition error (BSSE) of less than 0.05 eV at the EC(PBE0-PC) level of theory, so that we can neglect the BSSE in our analysis and we can compare the energetics between the periodic DFT within the plane-wave basis set and the EC(CASPT2-PC) within the localized basis set approaches. The spin multiplicity is specified in the RASSCF module of OpenMolcas that we use to compute the energy of the first five states. A defective cluster model is employed to simulate the doublet spin multiplicity of a superoxide-based system. This cluster is obtained by removing one Li atom from the stoichiometric system (thus leading to Li_2–*x*_O_2_ where *x* = 0.125), which corresponds to the removal of one electron and thus leaves a positively charged (+1) system. The convergence thresholds on total energy are 10^–5^ and 2 × 10^–5^ eV (10^–6^ Ha) for the PBC(PBE) and EC(CASPT2-PC) approaches, respectively.

## Results and Discussion

The oxygen release from the (112̅0) Li_2_O_2_ surface is first addressed via a rigid scan at the DFT-PBE level of theory in two coordinates: (i) the oxygen–oxygen bond length (*d*_O–O_) and (ii) the oxygen–surface distances (*d*_O–Surf_) (see Figure 1b). We are aware that, in principle, oxygen evolution can take place *via* more intricate paths including more variables, *e.g.,* surface reconstruction and molecule reorientation. While the detailed evaluation of overall geometrical rearrangements would require more demanding theoretical efforts (for example employing nudge-elastic band methods or molecular dynamic approaches), this first *ab initio* study on oxygen release from Li_2_O_2_ surfaces *via* the EC(CASPT2-PC) approach aims for simplicity and explores the potential energy surfaces (PESs) in two fixed reaction coordinates, inspired by the similar approach adopted by Pierini *et al.* to investigate the oxygen release from the LiO_2_^–^ trimer and unveil the nature of the ground state as function of the O–O distance.^42^ We generate the PESs by varying the dioxygen bond length from a maximum value, *i.e.*, the bond length of peroxide moiety (O_2_^2–^) lying in the topmost layer of the Li_2_O_2_ surface (*d*_O–O_ at the Li_2_O_2_ surface = 1.55 Å), and considering some representative values, such as the typical superoxide bond length (*d*_O–O_ in O_2_^–^ = 1.35 Å) and the ^3^O_2_/^1^O_2_ bond lengths from both DFT-PBE and experimental values (*d*_O–O_ in ^3^O_2_/^1^O_2_ DFT-PBE = 1.235 Å, *d*_O–O_ in ^3^O_2_ exp. = 1.2075 Å, *d*_O–O_ in ^1^O_2_ exp. = 1.215 Å).^56^ In particular, the following *d*_O–O_ values are explored, together with the oxygen–surface distances as a measure of the oxygen molecule departure from the Li_2_O_2_ surface (*d*_O–Surf_):

(i) *d*_O–O_ (Å): 1.205, 1.208, 1.210, 1.215, 1.220, 1.225, 1.230, 1.235, 1.250, 1.300, 1.350, 1.400, 1.450, 1.500, 1.550, 1.600
(ii) *d*_O–Surf_ (Å): 1.00, 1.15, 1.25, 1.50, 1.75, 2.00, 2.25, 2.50, 2.75, 3.00, 3.25, 3.50, 3.75, 4.00, 4.50, 5.00

**Figure 2:**
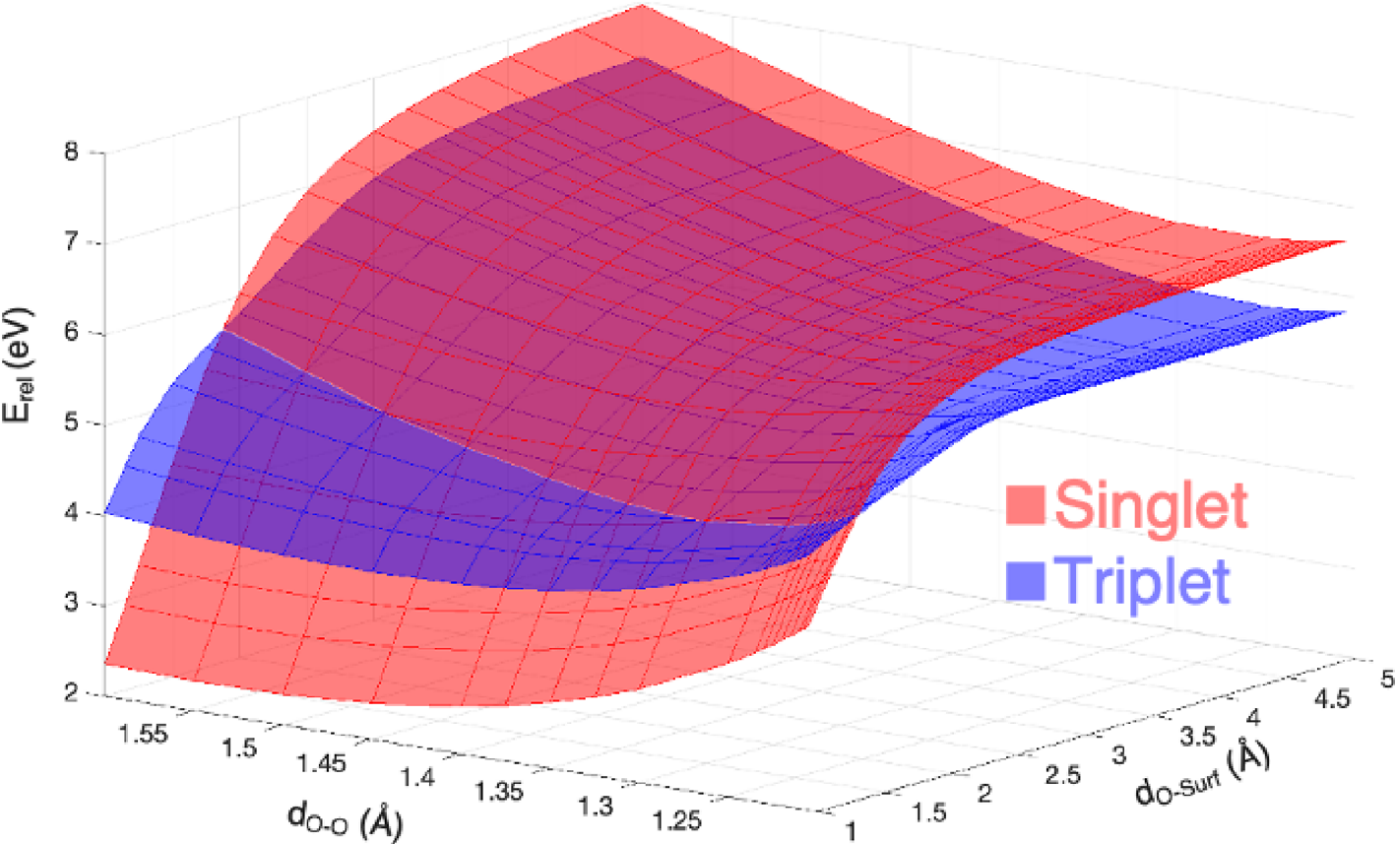
Singlet/triplet potential energy surfaces (PES) computed at the DFT-PBE level of theory for the oxygen release from the (112̅0) Li_2_O_2_ surface in the two coordinates. Relative energy of each point along the PES is calculated considering the energy of the (112̅0) Li_2_O_2_ surface as reference (*i.e.*, where the O_2_ moiety is still a peroxide lying in the topmost layer of the surface, *d*_O–Surf_ = 0.0 Å, *d*_O–O_ = 1.55 Å, *E*_rel_ = *E*_(d_O – Surf_; d_O – O_)_ – *E*_(0.0; 1.55)_).

Figure 2 shows the resulting PESs for the oxygen release obtained for the periodic model at DFT-PBE level of theory considering two different spin multiplicity for the whole system, *i.e.*, singlet and triplet (red and blue surfaces, respectively). The singlet/triplet state will be referred as S/T from this point forward, so that any point along the corresponding PES can be labeled as S/T-(*d*_O–Surf_; *d*_O–O_) indicating the singlet/triplet configuration of a given structure featuring the (*d*_O–Surf_; *d*_O–O_) pair of coordinates. Close to the surface (low *d*_O–Surf_ values), the whole system keeps the singlet multiplicity of the peroxide molecule, while triplet becomes the lowest energy state when the oxygen moiety is released up to ∼2 Å far from the surface. At this *d*_O–Surf_ value, we also find a reorganization of *d*_O–O_ from 1.55 to 1.35 Å, suggesting that the peroxide moiety stabilizes as superoxide. Magnetization moment of ∼0.4 on both oxygen atoms confirm that superoxide is formed at T-(2.00 Å; 1.35 Å). With increasing *d*_O–Surf_, further shortening of oxygen–oxygen bond length occurs from 1.35 to 1.25 Å, indicating that molecular rearrangement toward the molecular oxygen state is taking place. Far from the surface (*d*_O–Surf_ > ∼2.5 Å) and within the range of molecular oxygen (*d*_O–O_ = 1.25 Å), the S-PES lies ∼0.7 eV above the T-PES, and we can assess that DFT-PBE predicts oxygen to be released from the Li_2_O_2_ interface in its preferential triplet state. As already stated by Zaichenko *et al*.^41^ and Pierini *et al*.,^42^ the oxygen–oxygen bond length is a useful coordinate to follow the subsequent oxidation up to the final oxygen release and thus can be used as a simple descriptor to predict the formation of oxidation products.

Despite predicting the feasible oxygen release as a gradual removal from the peroxide interface, DFT is not able to keenly characterize the complex and highly correlated electronic states along the PES: our DFT results are obtained by applying constrains on spin multiplicity. Conversely, multireference methods, such as CASPT2, can access the electronic structure of radical species, such as O_2_^–^, and any elusive open- or closed-shell configurations by taking electronic correlation effects into account.^36−40^ By employing the electrostatic embedding approach to describe the extended surface slab as the environment, we are able to attain mechanistic insights into the release of singlet oxygen from the Li_2_O_2_ surface beyond DFT. From the surface slab, we carve up a small stoichiometric cluster (comprising 4 Li_2_O_2_ f.u. and including the releasing oxygen molecule) embedded in a point charge array that mimics the surface while still preserving the interaction with the remaining crystal lattice (see Figure 1b).

**Figure 3:**
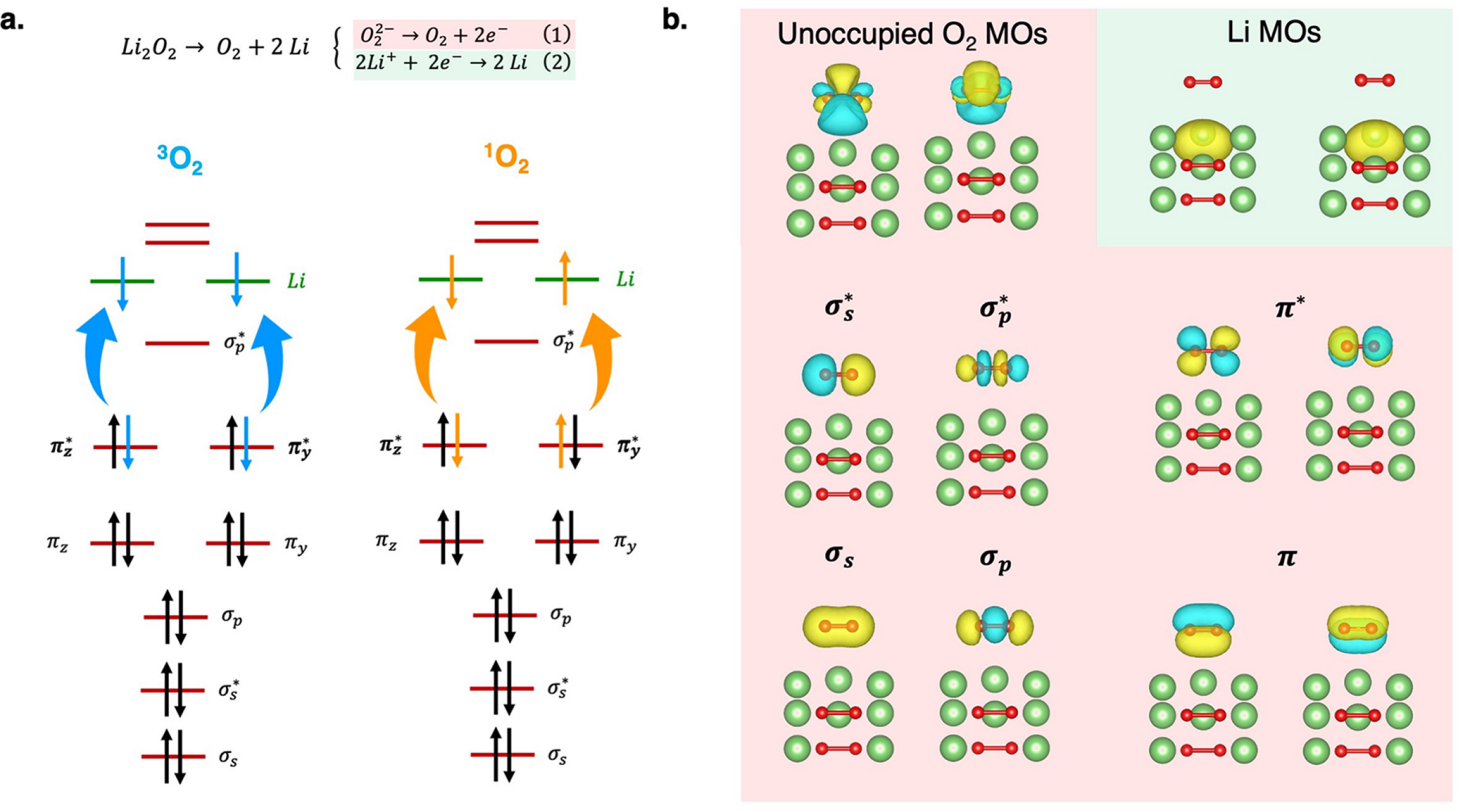
(a) Li-air battery charging process and ^3^O_2_/^1^O_2_ electronic configuration upon lithium peroxide oxidation (blue/orange arrows); (b) MOs considered in the CAS(14e,12o) active space. Color code as in Figure 1; isodensity positive and negative values (0.05 a.u.) are depicted in yellow and cyan, respectively.

Validation of the CASPT2 method requires the system definition in terms of the number of electrons and molecular orbitals, that is the complete active space (CAS(*n*_e_,*x*_m_)) within the CASSCF approach. In Table 1, we report CASPT2 calculations on the isolated triplet (^3^O_2_) and singlet (^1^O_2_) oxygen molecule with different active spaces. Convergence is evaluated in terms of both oxygen–oxygen bond length (*d*_O–O_) and triplet-to-singlet excitation energy (Δ*E*_S–T_) and is achieved with the active space CAS(12e,10o). The active space is then expanded with two extra electrons from two molecular orbitals (MOs) localized on the topmost Li atoms, thus giving the CAS(14e,12o). As shown in Figure 3, this active space comprises the most important MOs (σ_s_ σ_s_* σ_p_ π_y_ π_z_ π_y_* π_z_* σ_p_*) plus two unoccupied ones for the oxygen molecule and two Li orbitals. Such a converged CASSCF wavefunction is expected to reliably describe the Li-air battery charging process, thus avoiding the use of any spin constrains and allowing accurate predictions. With this active space, the PES for oxygen release is characterized by means of the embedded-cluster CASPT2 within the point charge field (*i.e.*, EC(CASPT2-PC) approach).

**Table 1:** Bond Length (*d*_O–O_) for Isolated Singlet (*d*_O–O,S_) and Triplet (*d*_O–O,T_) Oxygen Molecule and Excitation Energy (ΔE_S–T_) Calculated at CASPT2 Level of Theory with Different Active Spaces

	CAS(8e,6o)	CAS(10e,8o)	CAS(12e,10o)	CAS(12e,12o)
*d*_O–O,S_ (Å)	1.22	1.23	1.23	1.23
*d*_O–O,T_ (Å)	1.21	1.22	1.22	1.22
Δ*E*_S–T_ (eV)	0.95	1.24	1.04	1.02

**Figure 4:**
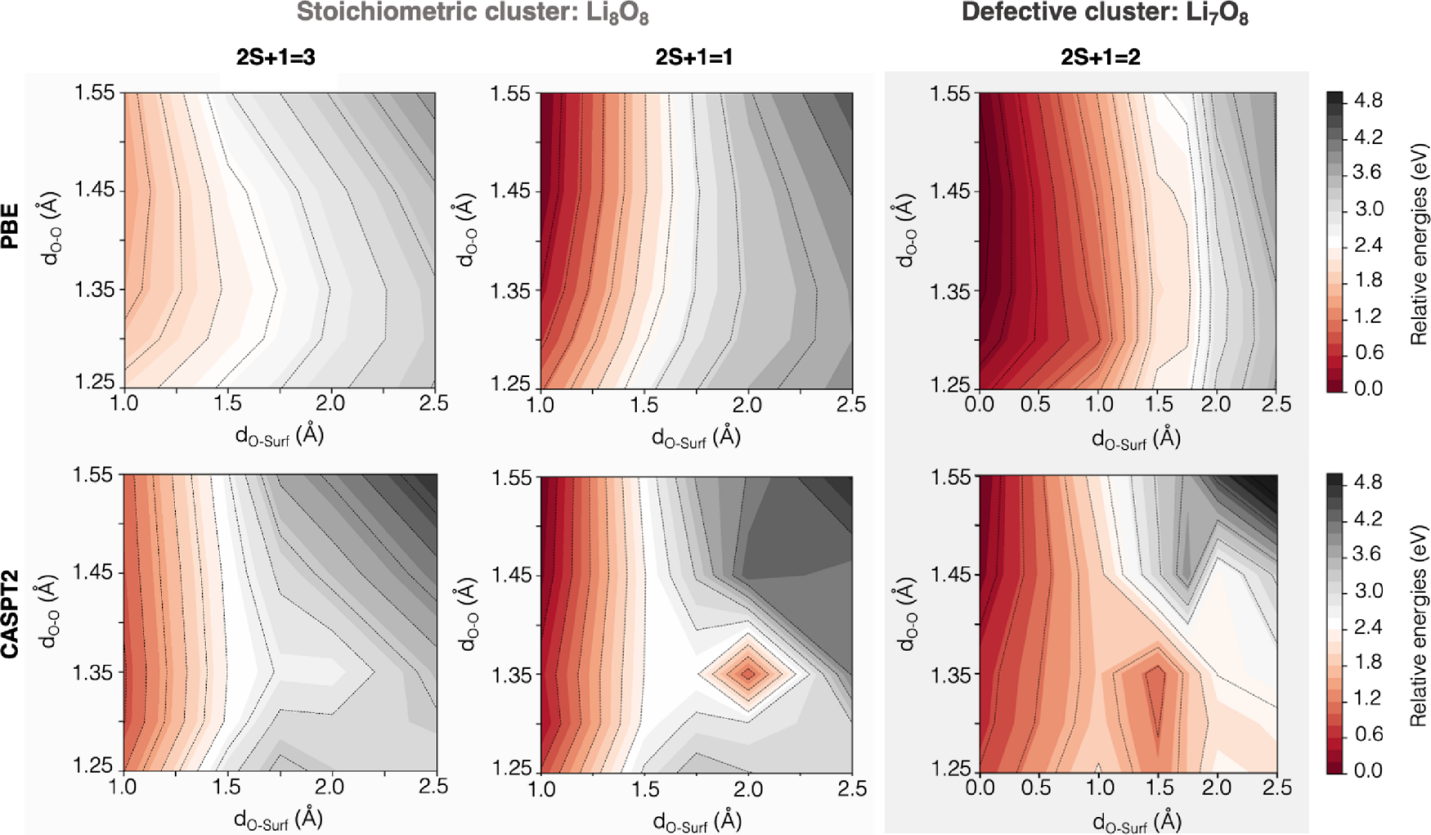
Color energy maps of oxygen moiety release from the Li_2_O_2_ surface computed at (top) PBC(PBE) and (bottom) EC(CASPT2-PC) levels of theory in (left) stoichiometric and (right) defective cluster. Different spin multiplicity is declared. Relative energies are referred to as *E*_1.00,1.55_ as defined in Figure 2.

The newly characterized PES for the oxygen release process from the Li_2_O_2_ surface is compared to the one obtained within periodic DFT (*i.e.*, the PBC(PBE) method, see top and bottom panels of Figure 4 for comparison). Again, the mechanism is unveiled by considering the two coordinates, but now only the most significant distance ranges (*d*_O–O_ ≈ 1.25–1.55 Å and *d*_O–Surf_ ≈ 1.00–2.50 Å). In general, CASPT2 shifts the relative energies to lower values, without altering the singlet/triplet trend. Similar outcomes are obtained close to the surface (low *d*_O–Surf_ values), where both PBC(PBE) and EC(CASPT2-PC) detect the oxygen moiety as a peroxide (*d*_O–O_ = 1.55 Å, see reddish areas in corresponding panels for lowest-energy states). Hereby, the system preserves the total singlet spin multiplicity of the lithium peroxide surface. When *d*_O–Surf_ increases, comparable shortening of *d*_O–O_ is detected by both methods, suggesting a general stabilization in a singlet superoxide state. According to the embedded-cluster CASPT2 results, a superoxide species is formed at S-(2.00 Å; 1.35 Å), which represents a local minimum along the S-PES and requires an energy barrier of ∼2.40 eV, in close agreement with previous outcomes on molecular systems.^41^ This local minimum is neither detected from the periodic DFT calculation (PBC(PBE) level of theory, see Figure 4 top panel) nor via electrostatic-embedded cluster approaches at PBE0 and CASSCF levels of theory (EC(PBE0-PC) and EC(CASSCF-PC) in Figures S1 and S2 in the SI, respectively). These results suggest that only incorporating both static and dynamic electron correlation effects *via* the CASPT2 method provides a reliable description of the subtle electronic features of these elusive radical superoxide species.

To represent the electronic state of a superoxide (O_2_^–^) and to characterize the nature of the energy barrier required for its formation, we consider a defective cluster as representative model for a positively charged system with a doublet spin multiplicity induced by removal of a Li atom (*i.e.*, the Li_7_O_8_ defective cluster). As a matter of fact, formation of Li vacancy in Li_2_O_2_ has been observed at a potential of 2.85 V, and some recent reports have highlighted Li removal as a significant step prior to superoxide formation.^45,57^ Modeling the EC(CASPT2-PC) PES in our Li-defective system can thus be representative for OER coupled to Li desorption. The active space for the charged model contains one less electron, leading to CAS(13e,12o). Corresponding PESs obtained at PBC(PBE) and EC(CASPT2-PC) levels of theory are displayed on the right side of Figure 4. Previous outcomes can be confirmed, including the presence of the stable superoxide, which is not visible in the PBC(PBE) PES. In this case, the superoxide is already released at 1.50 Å from the surface and provided a smaller energy barrier compared to the stoichiometric system (∼1.78 eV *vs* 2.40 eV).

**Figure 5:**
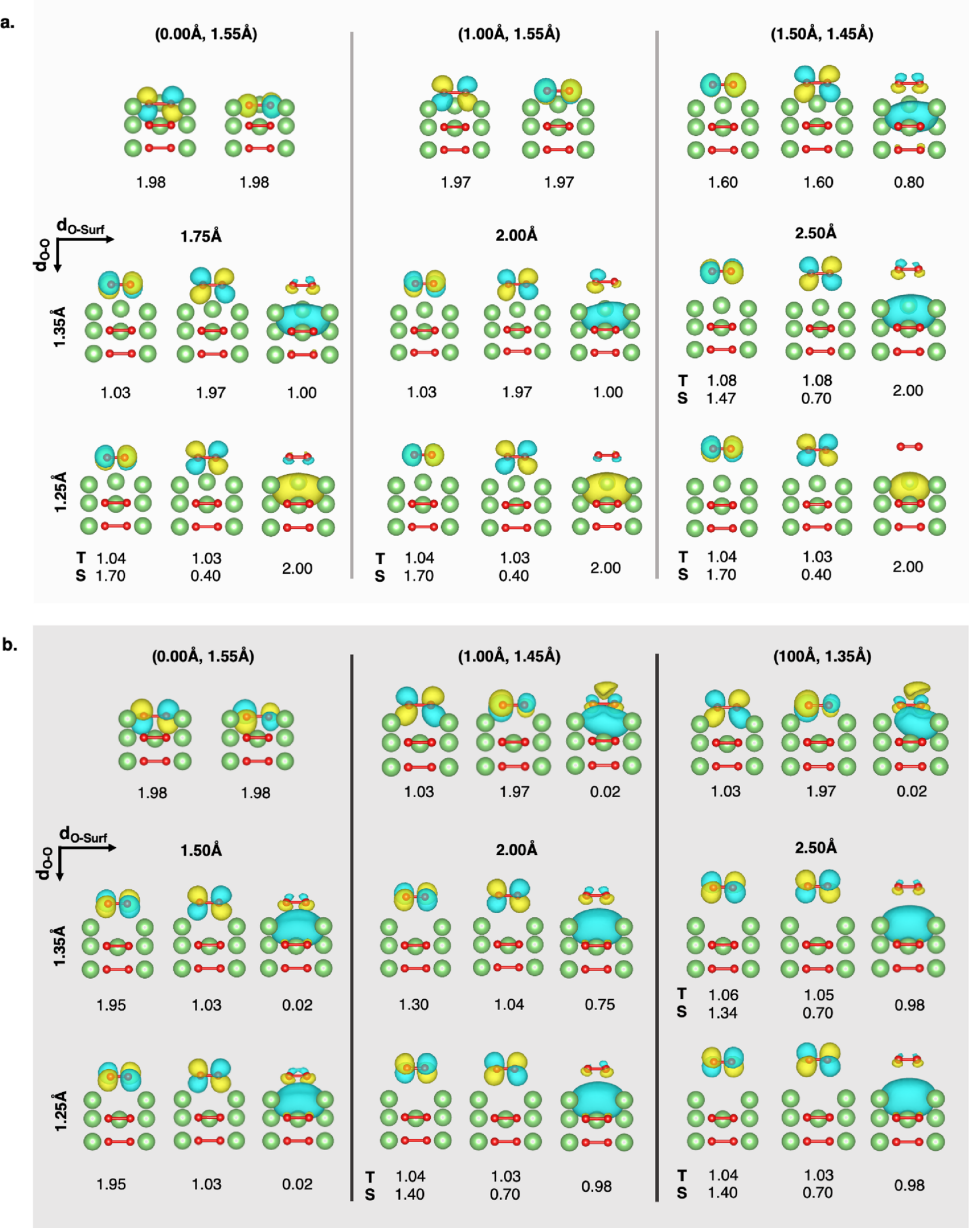
O_2_ π_y_*, π_z_* and Li MOs occupation for the most significant (*d*_O–Surf_; *d*_O–O_) points along the EC(CASPT2-PC) PES calculated for: (a) stoichiometric cluster in the CAS(14e,12o) active space; (b) defective cluster in the CAS(13e,12o) active space. Color code as Figure 3.

Detailed analysis of MOs occupation for each state along the PESs with different spin multiplicity helps unveiling the nature of each electron transfer step and thus the oxidation mechanisms. Figure 5 illustrates the O_2_ π_y_*, π_z_* and the Li MOs with corresponding occupation for some relevant points along the PES. By looking at the first panel in Figure 5a, the MOs occupation for the (1.75 Å; 1.35 Å) state suggests that in this configuration the peroxide is converted to superoxide (*i.e.*, π_y_*, π_z_* = 1.03, 1.97) *via* a one-electron oxidation, which is coupled to a local Li reduction (*i.e.*, Li MO = 1.00). The singlet spin state of such superoxide species is retained at 2.00 Å from the surface, but an additional oxidation occurs afterward: a second electron occupies the Li orbital (*i.e.*, Li MO = 2.00) when the departing superoxide reaches 2.50 Å from the surface or even at 1.75, 2.00, and 2.50 Å when the structural rearrangement to molecular oxygen configuration takes place, and the bond length shortens down to 1.25 Å. The second oxidation can either lead to a triplet (*i.e.*, π_y_*, π_z_* = ∼1.0, ∼1.0) or singlet (*i.e.*, π_y_*, π_z_* = ∼1.7, ∼0.4) state. This specific occupation of O_2_ π* MOs is ascribed to a closed-shell singlet configuration, which is also reported in a previous CASPT2 study on Li_2_O_2_ molecular systems to be the lowest energy state for singlet oxygen.^41^ The O_2_^2–^/O_2_^–^/O_2_ oxidation states are also confirmed by a Mulliken population analysis, with the negative charge of peroxide species becoming less negative when the oxygen moiety moves away from the surface (shifting from *q* = −0.5 as peroxide close to the surface, at (0.00 Å; 1.55 Å)/(1.00 Å; 1.55 Å), to *q* = −0.3 as superoxide far from the surface, at (1.75 Å; 1.35 Å)/(2.00 Å; 1.35 Å), and then up to *q* = 0.0 as molecular oxygen far away from the surface, at (1.75 Å; 1.25 Å)/(2.00 Å; 1.25 Å)/(2.50 Å; 1.25 Å)).

By looking at MOs occupation in the charged cluster (Figure 5b), we confirm that the releasing oxygen moiety is converted to superoxide, leading to the above-mentioned local minimum at (1.50 Å; 1.35 Å), but this first one-electron oxidation is coupled to a delocalized reduction involving several lithium atoms (*i.e.*, π_y_*, π_z_* = 1.95, 1.03 and Li MO = 0.02). This electron delocalization effect explains the lower occupation values obtained for Li MO compared to those reported for the stoichiometric cluster. Then, the second oxidation occurs at 2.00 Å from the surface with a localized character (*i.e.*, Li MO = ∼0.98), when molecular oxygen is released either as a singlet (π_y_*, π_z_* = ∼1.4, ∼0.7) or triplet (π_y_*, π_z_* = ∼1.0, ∼1.0) state. Understanding the relative stability of the predicted reaction intermediates is crucial to assess any potential strategy to limit the singlet oxygen release and favor an efficient reversibility within Li_2_O_2_ formation/decomposition upon battery cycling.

**Figure 6:**
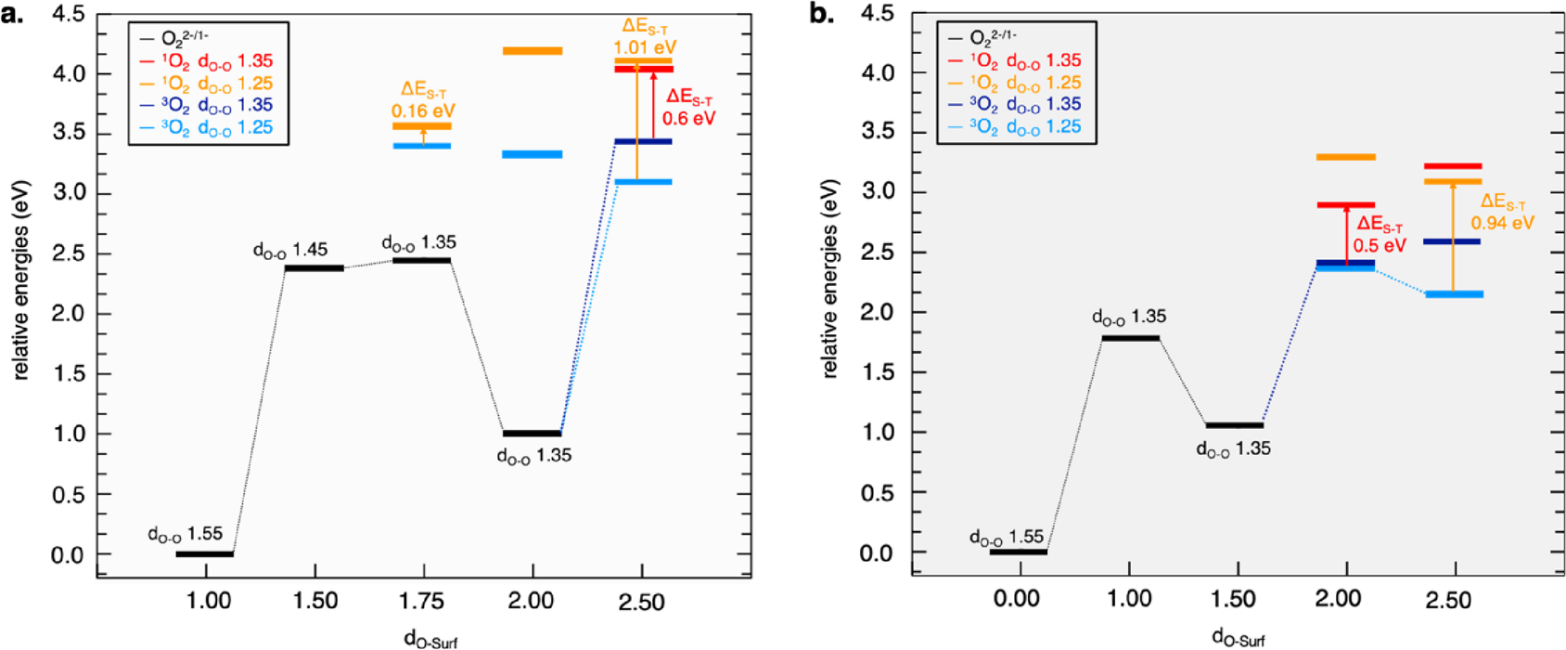
Energetics of singlet/triplet O_2_ release from (a) stoichiometric and (b) defective clusters by the EC(CASPT2-PC) method. Relative energies are referred to as *E*_1.0,1.55_ and *E*_0.0,1.55_. Color code is displayed in the graph.

In Figure 6, we report the energy plots of the EC(CASPT2-PC) PES as a function of the *d*_O–Surf_ coordinate for the stochiometric and defective clusters (panels a and b, respectively), in order to provide a general overview of the accessible mechanisms toward the ^1^O_2_ release. After forming the stable superoxide at 2.00/1.50 Å, further oxidation to molecular oxygen can take place at 2.50/2.00 Å from the surface, either preserving the superoxide bond length (1.35 Å, see blue lines in Figure 6) or upon structural reorganization (*d*_O–O_ dropping down to 1.25 Å, see cyan lines in Figure 6). The O_2_^–^ → O_2_ oxidation without bond reorganization, envisages ^3^O_2_ → ^1^O_2_ excitation energies (Δ*E*_S–T_) of ∼0.6 and ∼0.5 eV for stochiometric and defective clusters, respectively (red arrows in Figure 6a,b). The O_2_^–^ → O_2_ oxidation coupled to bond length shortening represents the favorite mechanism in both systems, with possible transition to ^1^O_2_, providing excitation energies of 1.01 and 0.94 eV for stoichiometric and defective clusters, respectively (orange arrows in Figure 6a,b). We can conclude that singlet oxygen is expected to arise from Li_2_O_2_ surface oxidation *via* formation of a stable intermediate superoxide with low extent (*i.e.*, *a* ∼ 2.0/2.5 eV energy barrier should be overcome) but can become easily accessible under battery operating conditions, when high voltage is supplied upon charge. It is worth mentioning that the ^1^O_2_ electronic configuration can also be reached at *d*_O–O_ = 1.35 Å, which represents an excited state along the PES of isolated molecular oxygen.^58^ This metastable state offers another available pathway for ^1^O_2_ release, as it would lie closer in energy to an isolated singlet oxygen. In addition, another reaction pathway in the stoichiometric cluster can be outlined: at ∼1.75 Å from the surface, a less stable superoxide species can be formed, and subsequent release of O_2_ can take place upon bond length reorganization (see corresponding cyan lines in Figure 6a). The energy difference between the initial peroxide (S-(1.0 Å; 1.55 Å)) and the ^3^O_2_ formed at 1.75 Å (T-(1.75 Å; 1.25 Å)) is ∼3.5 eV. Following this path, ^1^O_2_ lies only 0.16 eV above ^3^O_2_ (see corresponding orange arrows in Figure 6a). These results suggest that peroxide oxidation can also occur *via* a less stable superoxide species lying closer in energy to both ^3^O_2_ and ^1^O_2_ states.

To sum up, we suggest multiple reaction pathways leading to singlet oxygen as it would be released from the (112̅0) Li_2_O_2_ surface termination. One mechanism embodies the intermediate conversion to a stable superoxide and can be sketched as

(i) O_2_^2–^ => O_2_^–^ + e^–^ Δ*E*_1_ ≈ 1 eV
(ii) O_2_^–^ => O_2_ + e^–^ Δ*E*_2_ ≈ 2.5 / 2.0 eV without/with bond reorganization
(iii) ^3^O_2_ => ^1^O_2_ Δ*E*_3_ ≈ 0.6 / 1.0 eV without/with bond reorganization

giving an overall energy variation of ∼4 eV, regardless of any involved structural rearrangement. This path can be associated to the two-step one-electron oxidation process (see eqs 3 and 4) that is herein shown to be accessible *via* either the favorable triplet state with *d*_O–O_ = 1.25 Å or the metastable state with *d*_O–O_ = 1.35 Å. This probable ^1^O_2_ can still exert high reactivity related to its intrinsic excited state nature underlying the longer internuclear distance.^58^

Still, if oxidation occurs *via* the unstable superoxide, the following reaction scheme can be outlined:

(i) O_2_^2–^ => [O_2_^–^ + e^–^] = > O_2_ + e^–^ Δ*E*_1_ ≈ 3.4 eV
(ii) ^3^O_2_ => ^1^O_2_ Δ*E*_2_ ≈ 0.16 eV

with an overall energy variation of ∼3.6 eV for the direct conversion from peroxide to singlet oxygen, which clearly resembles the one-step two-electron process (see eq 1, reverse reaction). Whether it proceeds as two-step one-electron or one-step two-electron processes, the predicted energy variations are very similar (∼4 *vs* ∼3.6 eV, respectively), which is in line with experimental observation of singlet oxygen release occurring at voltages above ∼3.5 V and previous calculated barriers of ∼3.4 eV.^21,23,32,41^

Although the mechanistic outcomes from the stoichiometric and defective cluster models seem comparable, we should highlight that Li_2_O_2_ oxidation results to be favored on a defective (112̅0) surface. As already highlighted by previous *ab-initio* DFT study on Li_2_O_2_ surfaces, the concurrent lithium desorption upon charge is a rather significant process that can play a noninnocent role on oxygen release and underlying redox mechanisms.^45,57^ Another reaction scenario can be outlined as coupled to Li desorption:

(i) O_2_^2–^ => O_2_^–^ + e^–^ Δ*E*_1_ ≈ 1 eV
(ii) O_2_^–^ => O_2_ + e^–^ Δ*E*_2_ ≈ 1.5 eV without/with bond reorganization
(iii) ^3^O_2_ => ^1^O_2_ Δ*E*_3_ ≈ 0.5 / 0.94 eV without/with bond reorganization

with an overall energy variation of ∼3/3.4 eV depending on whether bond reorganization takes place. The lower energetics obtained for such mechanism correlates with the previously suggested hypothesis of OER occurring along with Li desorption from the Li_2_O_2_ surface. These findings further corroborate the need to include interfacial features for reliable mechanistic predictions.

Multiple reaction pathways toward singlet oxygen release can be possible, especially during battery charging in the high voltage range (above 3.5 V), where the singlet oxygen release at the cathode interface can be easily induced. Clearly, unveiling the reaction landscape can assist rational strategies aiming to mitigate the singlet oxygen release by altering the reaction energetics toward the desired products. Beyond the most popular strategies, including the choice of some specific solvent or surface structure combinations,^41,43^ controlling ^1^O_2_ from its source rather than quenching its generation can be more efficient. The use of redox mediators able to accelerate the relaxation of singlet-state complexes to triplet-state ones through an increased intersystem crossing rate has recently emerged as innovative and viable solution.^59,60^ Our theoretical investigation and the consolidated computational tools can assist future works aiming to address these effects on the unveiled reaction mechanisms and assess further design strategies.

## Conclusions

As highly promising technology for large-scale applications, including the automotive industry and electric transportation, Li-air batteries are at the forefront of global scientific efforts in the energy storage research field. The main bottleneck toward their deployment is the poor reversibility of crucial reactions occurring upon battery cycling that is the oxygen reduction/evolution reaction (ORR/OER) during discharge/charge. Lithium peroxide, Li_2_O_2_, is the most abundant discharge product, but its desired decomposition upon charge is actually affected by side reactions leading to degradation of electrolyte components. Release of singlet oxygen has emerged as the main cause of electrolyte damage owing to its high reactivity, with the thermodynamically unfavorable formation being accessible in the battery operating conditions (*i.e.*, within the high voltage range). Due to the elusive chemistry of ^1^O_2_, understanding the fundamental features behind singlet oxygen formation pathways represents a great challenge for both experiments and theory. Here, a thorough theoretical investigation on singlet/triplet oxygen release from the (112̅0) Li_2_O_2_ surface is presented with the objective of unveiling the redox reactions and the underlying mechanism by means of an effective computational strategy. Our main findings can be summarized as follows:

(i) The embedded-cluster CASPT2 method represents a better suited computational tool to describe the O_2_^2–^/O_2_^–^/O_2_ reactivity on the (112̅0) Li_2_O_2_ surface than standard periodic DFT.
(ii) The integrated analysis of EC(CASPT2-PC) PES for both ^1^O_2_ and ^3^O_2_ release in two coordinates (*d*_O–Surf_, *d*_O–O_) with MOs occupation reveals that O_2_^2–^/O_2_^–^/O_2_ evolution consists of subsequent electron transfers from the π_y_* and π_z_* MOs within dioxygen moiety and the Li MO. The first electron transfer leads to peroxide-to-superoxide oxidation and is coupled to lithium reduction, while the second one converts the superoxide to molecular oxygen in either triplet or singlet spin state. Similar analysis on a Li-defective cluster model shows that the first oxidation is coupled to a delocalized reduction on the surface, while local electron transfer takes place during molecular oxygen release.
(iii) We detect multiple reaction pathways toward singlet oxygen release that can be accessible in the high voltage range working as thermodynamic driving force: (i) a two-step one-electron oxidation for the peroxide-to-superoxide-to-oxygen process that is passing through the stable superoxide acting as a reaction intermediate and then releases molecular oxygen either at *d*_O–O_ = 1.25 Å or *d*_O–O_ = 1.35 Å (a metastable excited state with longer internuclear distance that is still highly reactive); (ii) a one-step two-electron oxidation for the direct conversion from peroxide to singlet oxygen *via* an unstable superoxide that lies much closer in energy to ^1^O_2_; (iii) an additional two-step one-electron is favored when coupled to Li desorption, thus confirming our choice of including the solid interface for reliable mechanistic predictions.

Overall, our results highlight that the high voltage applied to charge the Li-air/O_2_ cell can drive the peroxide-to-superoxide/molecular oxygen oxidation. Multiple mechanisms are unveiled at the EC(CASPT2-PC) level of theory and are associated to feasible release of singlet oxygen from the mostly present (112̅0) Li_2_O_2_ surface, with energy barriers that are accessible under battery operation. Whether it proceeds *via* a superoxide intermediate in two-step one-electron oxidation, directly to free oxygen in one-step two-electron oxidation, or coupled to Li desorption, specific design strategies aiming at reducing the occurrence of parasitic ^1^O_2_ will be key for exploiting the full potential of Li-air batteries.

## Supporting Information Available

Convergence tests for the surface energy of (112̅0)-Li_2_O_2_ surface and energetics of oxygen release from EC(PBE0-PC) and EC(CASSCF-PC) methods (PDF)
