# Supplementary material for: New Insights on Singlet Oxygen Release from Li-Air Battery Cathode: Periodic DFT Versus CASPT2 Embedded Cluster Calculations
Source: J Chem Theory Comput. 2023 Jul 11;19(15):5210–20. doi: 10.1021/acs.jctc.3c00393 (PMC10413853; doi:10.1021/acs.jctc.3c00393)
Supplement: Supplementary file 1 — ct3c00393_si_001.pdf [file ct3c00393_si_001.pdf]

SUPPORTING INFORMATION

New Insights on Singlet Oxygen Release from Li-Air Battery Cathode: Periodic DFT versus CASPT2 Embedded Cluster Calculations

Francesca Fasulo,[1] Arianna Massaro,[2] Ana B. Muñoz-García,[1,3] and Michele Pavone[2,3,*]

[1]*Department of Physics "E. Pancini", University of Naples Federico II, Napoli, Italy*

[2]*Department of Chemical Sciences, University of Naples Federico II, Napoli, Italy*

[3]*National Reference Center for Electrochemical Energy Storage (GISEL)-INSTM, Florence, Italy*

***Corresponding author:** *michele.pavone@unina.it*

Structural model of $(11\bar{2}0)$-Li_2O_2 surface

The number of layers for the $(11\bar{2}0)$-Li_2O_2 surface has been selected via a convergence test on surface energy as function of slab thickness. The surface energy, γ, can be calculated as:

$$\gamma = \frac{E_{slab} - NE_{bulk}}{2A} \quad \text{(S1)}$$

where A is the area exposed by the surface, E_{slab} and E_{bulk} are the total energies of the surface slab and the Li bulk, respectively, and N is the number of formula units contained in the surface slab. The corresponding results are collected in Table S1.

Table S1. Surface energy (γ) and bond length of topmost layer peroxide molecules for the $(11\bar{2}0)$ surface slab of 3 and 5 layers (3L and 5L, respectively).

	**γ (meV/Å²)**	**d_{O-O} (Å)**
**3L**	53.55	1.54
**5L**	52.89	1.54

The surface energy changes of 1 meV/Å^2 between 3 (3L) and 5 layers (5L)-slab models. Meanwhile, we chose a 2x2x1 supercell so that the distances between the periodic images of released molecules are ~15 Å and 10 Å along a and b axis. This model guarantees that the periodic images do not interact, making possible to study the oxygen evolution from Li_2O_2 as a localized process. For the same reason, we built up an embedded-cluster model with distances between the cluster and edges of the point charge array of ~8Å along the x and y, and with thickness of ~8Å. This array extension around the cluster can allow to mimic the periodic surfaces and achieve convergence on electronic and energetic features [S1].

Energetics of oxygen release via EC(PBE0-PC) approach.

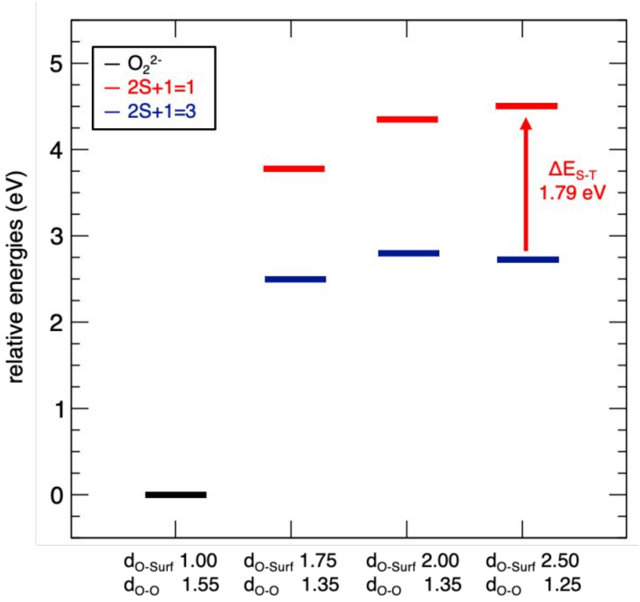

Figure S1. *Energetics of singlet/triplet O_2 release from stochiometric clusters by EC(PBE0-PC) methods. Relative energies are referred to $E_{1.0,1.55}$ and $E_{0.0,1.55}$, respectively. Color code is displayed in the graph.*

Color energy maps of oxygen moiety release via EC(CASSCF-PC) approach.

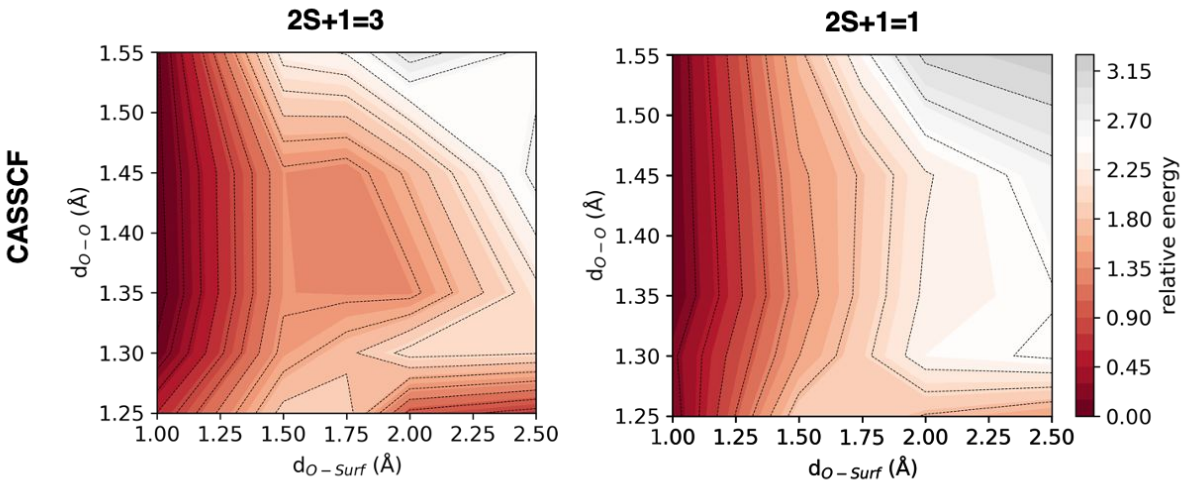

Figure S2. *Color energy maps of oxygen moiety release from Li_2O_2 surface computed at EC(CASSCF-PC) levels of theory in stoichiometric cluster. Different spin multiplicity is declared. Relative energies are referred to $E_{1.00,1.55}$ as defined in Fig. 2.*

References

[S1] Liao, P.; A. Carter, E. Optical Excitations in Hematite (α-Fe_2O_3) via Embedded Cluster Models: A CASPT2 Study. *J. Phys. Chem. C* **2011**, *115* (42), 20795–20805. https://doi.org/10.1021/jp206991v.
